# Psychotherapy and inhibitory control: Insights from fMRI research

**DOI:** 10.1111/pcn.70051

**Published:** 2026-03-26

**Authors:** Gioele Gavazzi, Ozge Ozkan, Viola Benedetti, Fabio Giovannelli, Valdo Ricca, Maria Pia Viggiano

**Affiliations:** ^1^ Department of Neuroscience, Psychology, Drug Research, Child Health University of Florence Florence Italy; ^2^ Psychiatry Unit, Department of Health Sciences University of Florence Florence Italy

**Keywords:** gyrus Cinguli, inhibition (psychological), meta‐analysis, prefrontal cortex, psychotherapy

## Abstract

**Aim:**

Despite the widespread clinical use of psychotherapy, the neural mechanisms linking treatment to changes in inhibitory control networks supporting self‐regulation remain unclear. This study addresses this gap by meta‐analyzing neuroimaging research on how psychotherapy affects brain regions involved in inhibitory control.

**Methods:**

We conducted a systematic literature review, selecting functional magnetic resonance imaging (fMRI) studies that examined inhibitory control tasks before and after psychotherapeutic interventions. Fifteen studies met the inclusion criteria and were analyzed using the activation likelihood estimation (ALE) algorithm.

**Results:**

Results revealed three significant clusters of activations, namely: the superior and medial frontal gyrus (including pre‐supplementary motor area), anterior cingulate cortex (ACC), and subcortical regions, such as the thalamus and globus pallidus. These areas are commonly reported as key nodes of the inhibitory control brain network.

**Conclusion:**

Overall, our main result indicates that psychotherapy might modulate key brain region activations involved in inhibitory control, particularly the prefrontal cortex‐anterior cingulate network. This suggests that psychotherapy enhances cognitive and emotional regulation through strengthening inhibitory mechanisms. Moreover, the observed neural changes appear to parallel those induced by pharmacologic interventions, pointing to potential convergent therapeutic targets despite differing mechanisms of action.

Executive functions comprise a range of high‐level cognitive abilities that support goal‐directed behavior, adaptation to complex and new situations, and regulation of social interactions.^1^, ^2^ The impact of executive functions on mental health, physical health, and overall quality of life emphasizes the importance of exploring the mechanisms of these functions to ensure well‐being.^3^ One of the most important executive functions is inhibitory control. Inhibitory control can be defined as the ability to control attention, behavior, thoughts, and emotions in order to override a strong internal predisposition or external lure.^4^

Response inhibition is the ability to suppress improper actions and impulses, is closely linked to numerous neurological and psychiatric conditions, as well as a wide range of behavioral and health problems.^5^ To assess these functions a variety of tasks are employed. Although these tests may vary from one study to another, it is generally assumed that each task assesses a common or closely related inhibitory mechanism. The most widely used tasks to probe response inhibition include the Go/No‐Go task, the Stop‐Signal task, and the Stroop task.^5^, ^6^, ^7^, ^8^ Previous studies using these behavioral protocols have demonstrated strong associations between inhibitory control deficits and several major psychiatric disorders, including substance use disorder (SUD), schizophrenia, bipolar disorder (BD), depression, obsessive‐compulsive disorder (OCD), anxiety disorders, post‐traumatic stress disorder (PTSD), attention deficit hyperactivity disorder (ADHD), borderline personality disorder (BPD), cerebral autosomal dominant arteriopathy with subcortical infarcts and leukoencephalopathy (CADASIL).^9^, ^10^, ^11^, ^12^, ^13^, ^14^, ^15^, ^16^, ^17^, ^18^ Functional magnetic resonance imaging (fMRI) studies have identified specific brain regions associated with impairments in inhibitory control, providing insights into the neural mechanism underlying these disorders. For instance, a meta‐analysis revealed abnormal brain activation in patients with severe psychiatric conditions, particularly in the bilateral cingulate gyri and bilateral medial frontal cortices (e.g. the medial portion of the superior frontal gyrus).^19^ Response inhibition has been associated with both pharmacologic and psychological treatment effects in certain disorders, showing correlations with cortical activity modulated according to treatment outcomes.^20^, ^21^

Among psychotherapeutic approaches, Cognitive Behavioral Therapy (CBT) stands out as one of the most extensively studied and evidence‐based methods. By modifying neural circuits, CBT contributes to improvements in response inhibition, symptom reduction, and lowering relapse risk in severe psychiatric disorders, as demonstrated in fMRI studies.^22^, ^23^, ^24^, ^25^ Several neuroimaging studies have demonstrated significant changes in inhibitory control network activation following CBT.^26^ However, despite advancements in neuroscience, the neurobiological mechanisms underlying psychotherapy are still not fully understood.^27^ To deepen this relationship, the impact of CBT on inhibitory control has been examined across various disorders, with a particular focus on each condition. For instance, a study on PTSD patients investigating the neural effects of CBT reported changes in the bilateral dorsal anterior cingulate cortex (dACC), right superior frontal gyrus, and premotor cortex.^28^ Similarly, another study on PTSD and major depression (MD) found that patients exhibited additional brain regions associated with CBT effects, including the dorsal anterior cingulate, middle cingulate, thalamus, and striatum.^29^ Studies on OCD patients have identified overlapping regions, while also pointing to further involvement of the left medial and right middle frontal gyri, and the left posterior and right middle cingulate gyri.^30^, ^31^ Additionally, in patients with SUD, altered brain activity related to response inhibition was observed in the thalamus, anterior cingulate gyrus, the middle and superior frontal gyri, and temporal gyri.^9^, ^32^

Several brain regions have shown inconsistent results across studies. To address this, we meta‐analyzed fMRI studies across a diverse range of disorders. This approach provides a progressive opportunity to identify a generalizable neural network underlying inhibitory control recovery, rather than focusing solely on disorder‐specific changes. Although studies in this area are steadily increasing, this is the first meta‐analysis on the effects of psychotherapy on inhibitory control using fMRI. Our findings provide some new insights into the understanding of the neurobiological mechanisms underlying psychotherapy, which may guide its application and development in practice.

## Methods

### Search strategy and selection criteria

**Fig. 1:**
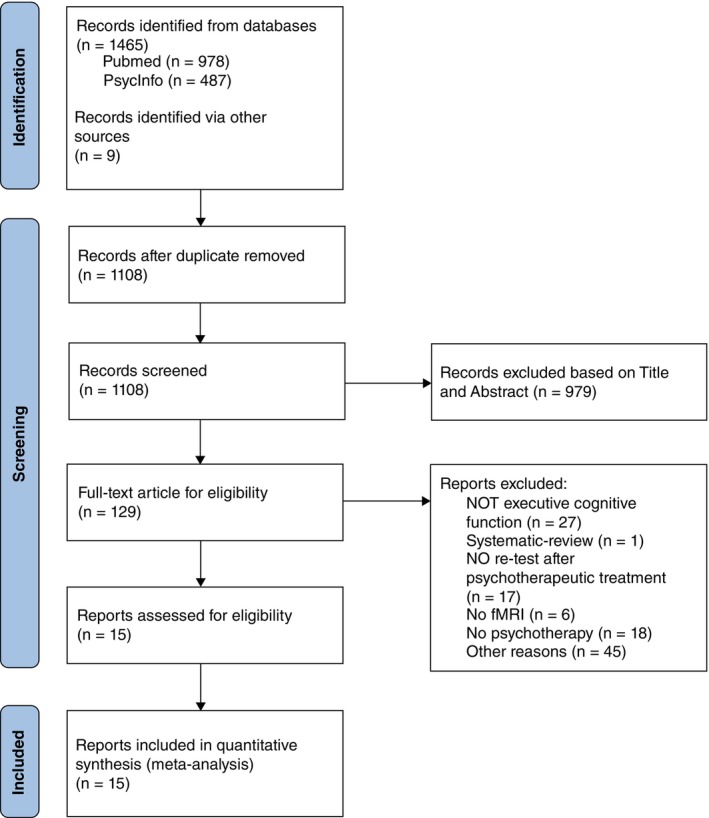
PRISMA flow chart. PRISMA 2020 flow diagram for new systematic reviews which included searches of databases, registers, and other sources.

We conducted a systematic and comprehensive literature search to select relevant fMRI studies published up to 4 May 2024 using the databases PubMed (https://pubmed.ncbi.nlm.nih.gov/ —accessed on 4 May 2024) and PsycInfo (https://www.apa.org/pubs/databases/psycinfo; accessed on 4 May 2024). To build our string or research we used MeSH Terms (Pubmed), Controlled Vocabulary (PsycInfo thesaurus with explode function). The selected keywords were combined using the Boolean operators AND and OR. The search input was the same both for Pubmed and PsycInfo: (‘Psychotherapy’ OR ‘Psychotherapy*’) AND (‘Functional Neuroimaging’ OR ‘Functional Magnetic Resonance Imaging’ OR ‘fMRI’) AND (‘Executive Function*’ OR ‘Cognitive Control’ OR ‘Inhibit*’ OR ‘Response Inhibition’ OR ‘Inhibitory Control’ OR ‘Impulse* Control’ OR ‘Self‐control’ OR ‘Cognitive Inhibition’ OR ‘Action Inhibition’). Additional studies were searched from the references of all identified publications. Eligibility was determined *via* a two‐step procedure performed by three of the authors (OO, VB, and GG). First, the titles and abstracts of all identified articles were screened. In the second step, the full texts of studies were independently examined according to predefined eligibility criteria, and agreement was reached after discussion. No inclusion criteria were modified retrospectively during the selection procedure. Our study was not pre‐registered and was conducted following the preferred reporting items for systematic reviews and meta‐analyses (PRISMA) guidelines.^33^ The studies were considered eligible if they met the following inclusion criteria: (1) whole‐brain analysis performed on fMRI data (e.g. we excluded studies conducted using positron emission tomography). Inhibitory control paradigms rely on rapid, event‐related task contrasts to elicit response inhibition, which are well captured by the temporal resolution of the BOLD signal but are not adequately or comparably measured using positron emission tomography (PET imaging); (2) availability of coordinates of activation foci clearly provided either in Montreal Neurological Institute (MNI) or Talairach reference space; (3) studies conducted on adults who performed, before and after a psychotherapy treatment, an inhibitory task in fMRI or were measured a correlational analysis between BOLD activations and an inhibitory control task. Exclusion criteria: studies involving children or the elderly studies including individuals with neurological conditions; studies using nonstandard neuroimaging data analysis or procedures. Additionally, all studies using region‐of‐interest analyses based on *a priori* defined regions, without an initial whole‐brain analysis, were systematically excluded. Applying these strict criteria allowed us to select homogeneous studies, thereby obtaining more robust measures.^34^ In particular, by trying to maximize homogeneity while ensuring sufficient statistical power, study selection prioritized the inclusion of well‐defined inhibitory control contrasts rather than the specific type of psychotherapy. Although the included studies varied in therapeutic approach and experimental paradigms, they shared a common focus on conflict processing and response inhibition.Which are the crucial processes directly aligned with the neural systems targeted in the present meta‐analysis. This strategy is consistent with established recommendations for coordinate‐based neuroimaging meta‐analyses, which emphasize task and contrast homogeneity over intervention labels to enhance interpretability and robustness of results^35^. See Fig. 1 for the PRISMA flow chart.

As shown in Fig. 1 of the PRISMA flow chart, following the elimination process, 15 studies were included in the meta‐analysis. Data, including coordinates, sample size, demographic details, type of psychotherapeutic intervention, and the type of inhibitory task used, were collected independently by two of the authors (O.O. and G.G.). Any disagreements between the reviewers were resolved through discussion and consensus with a third reviewer (V.B.). No automation tools were used in the data collection process. When result coordinates or critical study details were missing or unclear, the specific missing data points (e.g. a particular coordinate contrast) were excluded from the analysis. Details of the included studies are provided in Supplementary Table S1.

### Clinical and behavioral analysis

*P*‐values from studies reporting quantitative inferential statistics were evaluated using Fisher's combined probability method. Using this approach, we analyzed both the *P*‐values derived from pre‐ *versus* post‐intervention comparisons of clinical outcomes and those associated with reported task's inhibitory measures. For studies reporting threshold values (e.g. *P* < 0.001), a conservative estimate of *P* = 0.001 was adopted. Studies that did not report *P*‐values were excluded from the combined analysis. Consequently, 12 of the 15 eligible studies were included in the analyses.

### Activation Likelihood Estimation (ALE)

All coordinates were analyzed in MNI space. Coordinates originally reported in Talairach space were converted to MNI space using the Brett transformation and were subsequently analyzed using the activation likelihood estimation (ALE) meta‐analysis algorithm implemented in GingerALE version 3.0.2 (www.brainmap.org/ale). ALE is a coordinate‐based meta‐analysis method that uses peak coordinates from functional studies as inputs and has been well described in previous methodological papers.^35^, ^36^ The ALE algorithm evaluates the convergence of activation foci from the included neuroimaging studies, modeled as probability distributions, against null distributions of random spatial associations while controlling for sample size. To minimize within‐experiment effects, the non‐additive algorithm described in Turkeltaub^37^ was applied. Inference was made at the cluster level, as this provides the best balance between sensitivity and specificity.^35^

A voxel‐level threshold of *P* < 0.005 and a cluster‐level family‐wise error (FWE)–corrected threshold of *P* < 0.05, based on 2.000 permutations, were applied. The assessed studies are comprehensively documented in the Supplementary Table S1.

The neuroanatomical coordinates reported in Talairach^39^ space were transformed to MNI space for all analyses. Whole‐brain maps of the thresholded ALE images were visualized in Mango V.4.0.1 (http://rii.uthscsa.edu/mango/), an anatomical image overlay program, superimposed onto a standardized anatomical template.

## Results

### Results of the study search

A PRISMA flow chart of the article selection process is illustrated in Fig. 1. A full‐text review of these works initially identified 1108 potentially eligible studies. After evaluating of the full text of these articles, 129 studies were potentially eligible. Following a careful assessment, data were ultimately extracted from 15 studies that reported results related to contrast associated with an inhibitory process before and after a psychotherapeutic treatment (Supplementary Table S1). The main characteristics of the studies included in the analysis are reported in the Supplementary Table S1.

### Sample characteristics, clinical and behavioral results

The present meta‐analysis was based on 15 included studies, comprising a total of 313 participants. The weighted mean age of the overall cohort was calculated as 32.25 years based on the 297 participants for whom age data were available. The sample included adult patients diagnosed with OCD, SUD, Panic Disorder, MDD, PTSD, and other psychiatric conditions. All studies targeted an inhibitory control mechanism: Stroop task (8 studies), Go/No‐go (4 studies), or Stop Signal Task (3 studies) paradigms. The therapeutic interventions and their durations slightly varied across the sample. The majority of the interventions (9 studies) were CBT‐based, with durations typically spanning from 8 to 16 weeks. The analysis also incorporated Cognitive Remediation Therapy (2 studies; one 3‐week and one 12‐week protocol), short‐term psychodynamic inpatient treatment (1 study, 4 weeks), Transference‐Focused Psychotherapy (1 study, 9–10 months), Psycho‐educational and Cognitive Behavioral Stabilizing Group Treatment (1 study, 20 weeks), and a Psychoeducation Program (1 study, 12 weeks). Relative to baseline symptoms, participants who received Psychotherapy demonstrated statistically significant clinical improvements in 13 of the 15 studies reviewed.

Regarding the efficacy of psychotherapeutic treatments, Fisher's combined probability method yielded a test statistic of *X* ^2^ = 133.66, corresponding to a global *P*‐value <0.0001. This result indicates overwhelming evidence against the global null hypothesis that all studies reflect chance findings, supporting a robust and systematic improvement associated with the treatment.

For what concerns inhibitory control, three studies did not report any results. Of the remaining 12 studies, only one showed no improvement, four reported *P*‐values close to significance, and seven demonstrated statistically significant improvements. These detailed characteristics are provided in Supplementary Table S1.

Fisher's combined probability test (*n* = 12) yielded a statistic of *X* ^2^ = 42.98, corresponding to a global *P*‐value <0.01. This result indicates statistically significant evidence that inhibition improves after the treatment if compared against the global null hypothesis.

### ALE results

**Fig. 2:**
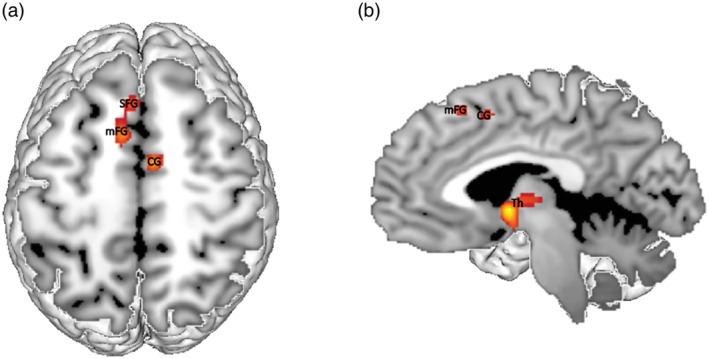
ALE meta‐analysis map for the groups of our data selection. The algorithm converged on a cluster of 12,696 mm^3, encompassing the superior frontal gyrus (BA8), the medial frontal gyrus (BA6) anterior and medial cingulate gyrus (BA24). *P* < 0.05 cluster‐level corrected inference using *P* < 0.005 uncorrected at voxel‐level as the cluster‐forming threshold. BA, Brodmann area.

The ALE meta‐analysis of the studies collected (Fig. 2 and Table 1) identified three clusters. The first cluster (1400 mm^3) had three peaks; it was centered in the thalamus and included the medial dorsal nucleus, the lentiform nucleus, and a portion of the globus pallidus. The second cluster (1272 mm^3) was characterized by 2 peaks and encompassed both the medial frontal gyrus (mFG‐BA6) and the cingulate gyrus (CG). Finally, we observed a cluster (1264 mm^3) composed of 2 peaks located in the anterior cingulate cortex (ACC) and in the superior frontal gyrus (SFG) (BA8).

**Table 1:** Results of ALE meta‐analysis

Cluster #	*x*	*y*	*z*	ALE	*P*	Z	Label (Nearest Gray Matter within 5mm)	Contributors
1	−2	−2	0	0.016168652	3.0128833E‐6	4.525,492,7	Left Cerebrum. Sub‐lobar. Thalamus. Gray Matter.	Yang *et al*. 2018; Lillevik Thorsen *et al*. 2020; DeVito *et al*. 2017
1	−2	−8	4	0.010482294	2.7058081E‐4	3.459,509	Left Cerebrum. Sub‐lobar. Thalamus. Gray Matter.	
1	−6	−16	4	0.0094595365	5.6595745E−4	3.255,494,6	Left Cerebrum. Sub‐lobar. Thalamus. Gray Matter. Medial Dorsal Nucleus	
2	6	−4	50	0.012383579	6.987672E‐5	3.808,605,4	Right Cerebrum. Frontal Lobe. Medial Frontal Gyrus. Gray Matter. Brodmann area 6	Lillevik Thorsen *et al*. 2020; DeVito *et al*. 2017; Perez *et al*. 2016
2	12	−4	42	0.011894586	1.0039186E‐4	3.718,029,3	Right Cerebrum. Limbic Lobe. Cingulate Gyrus. Gray Matter. Brodmann area 24	
2	10	8	42	0.009240487	7.061417E‐4	3.192,128,7	Right Cerebrum. Limbic Lobe. Cingulate Gyrus. Gray Matter. Brodmann area 24	
3	−8	10	48	0.013171922	3.80384E‐5	3.956,436,4	Left Cerebrum. Limbic Lobe. Cingulate Gyrus. Gray Matter. Brodmann area 24	Yang *et al*. 2018; Lillevik Thorsen *et al*. 2020; DeVito *et al*. 2019; Perez *et al*. 2016; Thomaes *et al*. 2012
3	−4	22	50	0.009754522	4.4972496E‐4	3.320,224,8	Left Cerebrum. Frontal Lobe. Superior Frontal Gyrus. Gray Matter. Brodmann area 8	

*Note*: Foci are reported as MNI coordinates.

Abbreviations: ALE, activation likelihood estimation; MNI, Montreal Neurological Institute.

## Discussion

Although psychotherapy is widely used in clinical practice, its effects on the brain remain under‐explored, particularly in terms of the neural correlates involved. While substantial research has examined the biological impact of pharmacotherapy, the neurobiological mechanisms underlying psychotherapy have received comparatively little attention. In this study, we aimed to address this gap by meta‐analyzing fMRI studies exploring the brain regions modulated by psychotherapy, focusing on tasks that engage inhibitory control. We gathered data from 15 studies where psychotherapy treatment improvements were also assessed through an inhibitory task. At the behavioral level, we observed that psychotherapy treatments provided significant improvements for symptomatic relief and improvements in behavioral measures of inhibitory control. For what concerns the associated brain activations, our ALE metanalysis algorithm converged in the superior frontal gyrus (SFG) and medial frontal gyrus (mFG), including the pre‐supplementary motor area (pre‐SMA) and the anterior cingulate cortex (ACC), the Globus Pallidus and the Thalamus. This finding indicates that psychotherapy can modulate key brain regions implicated in inhibitory control and that such modulation is related to improvement in clinical symptoms through enhanced inhibitory functioning.^22^, ^40^, ^41^

Recent and old fMRI meta‐analyses have highlighted these brain areas as crucial for inhibitory control.^42^, ^43^ For instance, a seminal meta‐analysis by Simmonds et al.^44^ demonstrated robust engagement of the pre‐SMA and dorsal ACC across response inhibition paradigms. While more recent meta‐analyses have further confirmed the central role of these regions and refined their functional organization during inhibitory control, showing a graded recruitment of the prefrontal cortex (PFC) along an inferior‐to‐superior axis as inhibitory demands increase, with higher‐demand conditions engaging progressively more superior prefrontal cortices.^42^, ^43^ Importantly, converging metanalytic evidence also indicates that these prefrontal regions support a domain general inhibitory control across both motor and cognitive domains. In particular, Apsvalka et al.^45^ demonstrated that the PFC dynamically targets task relevant representations to exert inhibitory control over both action and thought, providing a unifying framework for understanding how similar neural substrates are recruited across diverse inhibitory demands. An interpretation further supported by Anderson et al.,^46^ who conceptualize inhibitory control as a domain‐general regulatory mechanism mediated by medial and lateral superior prefrontal networks. By enabling the regulation of both thoughts and emotions, this mechanism provides a plausible neurocognitive pathway through which psychotherapeutic interventions may achieve clinical improvements. Notably, our analysis did not reveal the involvement of the very frequently reported brain region: the inferior frontal gyrus (IFG). The absence of IFG is noteworthy, considering it has been reported to be a key node in action stopping and inhibitory control models.^47^, ^48^ However, more recent studies suggest that the right IFG, rather than directly implementing reactive stopping, appears to be preferentially involved in proactive components of inhibition, such as anticipatory control settings, attention monitoring, and the preparation of inhibitory processes. In fact, meta‐analytic and lesion‐based evidence support the role of the right IFG in the proactive phase of cognitive control.^42^, ^49^ Within this framework, our pattern of results may indicate that psychotherapy interventions may preferentially enhance reactive inhibitory processes (that is: those responsible for the rapid implementation of behavioral suppression) rather than proactive control mechanisms. Consistently with this idea, our metanalysis revealed at the subcortical level the Globus Pallidus and the Thalamus. In fact, reactive control improvements would be expected to manifest at subcortical gating nodes within cortico‐striato‐thalamo‐cortical (CSTC) loops, including the globus pallidus and thalamus, which are critically involved in translating cortical control signals into effective behavioral inhibition.^50^, ^51^ This interpretation offers a parsimonious account of the presence of robust subcortical effects alongside the absence of IFG findings, suggesting that psychotherapy‐related change may also operate downstream of classical frontal control inhibitory regions.

Hence, the brain regions identified in the present study may play a central role in top‐down regulation and cognitive control, and their dysfunction has been linked to various psychiatric disorders characterized by impaired inhibitory processes, such as anxiety, depression, and obsessive‐compulsive disorder (OCD). The inclusion of a wide clinical spectrum in the present study allows for generalization across diagnoses, rather than being confined to disorder‐specific changes. Thus, the observed changes in PFC and ACC activity may suggest that psychotherapy enhances the capacity for inhibitory regulation, regardless of the clinical diagnoses, potentially resulting in improved emotional and behavioral control in clinical populations. This relationship between psychotherapy and the inhibitory control mechanisms aligns with the broader view of psychotherapy as a process that strengthens cognitive and emotional regulation through repeated, structured engagement with challenging psychological tasks. Indeed, the link between psychotherapy and the possible modulation of inhibitory circuits can be traced to the theoretical underpinnings of cognitive‐behavioral and emotion‐focused therapies, which emphasize altering maladaptive thought and behavioral patterns. Various studies support the idea that psychotherapy can induce significant changes in brain activation patterns, especially in regions associated with inhibitory control, such as the PFC and ACC^17^, ^52^ These approaches inherently rely on inhibitory mechanisms, such as suppressing automatic negative thoughts and overriding habitual responses, as widely shown in the literature.^22^ Neuroplasticity studies offer a plausible speculation: the repeated engagement of inhibitory processes during therapy sessions might strengthen synaptic connections within the PFC‐ACC network, thereby improving its functional connectivity and efficiency. Additionally, our results raise the hypothesis that psychotherapy may influence inhibitory control disorders by modulating cortico–striato–thalamo–cortical circuits beyond frontal cortical regions. In fact, the inhibitory control is implemented by means of these distributed circuits, where thalamic projections to frontal areas seem to play a crucial role in disorders of impaired inhibition, as shown by the clinical efficacy of deep brain stimulation targeting the anterior limb of the internal capsule.^53^, ^54^

Pharmacologic and psychological interventions, despite their different nature, frequently modulate activity in similar brain regions. For example, it is well known that antidepressants stimulate connectivity between the PFC and ACC, albeit through distinct molecular routes.^55^, ^56^, ^57^ On the other hand, both pharmacotherapy and psychotherapy, especially CBT, can target similar neural correlates while producing their effects through different mechanisms. As shown in a previous study^58^, one approach may increase connectivity between the cingulate cortex and somatosensory/motor regions, whereas the other may lead to a reduction in this connectivity. Notably, opioid signaling plays a crucial role in modulating inhibitory transmission within the PFC, influencing neural circuits associated with the therapeutic effects of psychotherapy.^59^ A speculative feature of this study is the integration of our findings with existing literature to highlight these brain regions as potential shared neural substrates for different therapeutic modalities.

On one hand, psychotherapy has been supposed to increase the ‘reflective pathway’ of emotional processing. This represents a ‘top‐down’ mechanism, focusing on emotional awareness that exerts cognitive control over emotional responses and dampens limbic hyperactivation.^60^, ^61^ On the other hand, pharmacotherapy primarily produces ‘bottom‐up’ modifications by directly targeting lower brain regions (i.e. brainstem, insula, and subgenual cingulate) and chemical messengers to regulate emotional reactivity.^62^ The integration of ‘top‐down’ and ‘bottom‐up’ processes offers a compelling neurobiological explanation for the benefits of combination therapy, suggesting a temporal synergy where rapid symptom relief by pharmacotherapy. According to literature, this approach would increase the brain's ‘plasticity window,’ facilitating the network reorganization driven by psychotherapy.^58^, ^63^, ^64^, ^65^

Although achieved through markedly distinct processes and mechanisms, a primary shared goal of the two therapies (psychotherapy and pharmacotherapy) appears to be the modulation of excitatory/inhibitory homeostasis in dysfunctional regulatory systems. A clear example of this is the gamma‐aminobutyric acid (GABA) system, the brain's principal inhibitory neurotransmitter, which is consistently disrupted in psychiatric disorders.^66^, ^67^ While pharmacotherapy acts directly on GABAergic mechanisms, psychotherapy may influence brain function indirectly by engaging higher‐order regulatory processes that support adaptive inhibitory control.

Although our findings are promising, several limitations should be acknowledged. First, the number of included studies was slightly below the recommended threshold (15 instead of 17), and this limitation was probably offset by the high methodological and conceptual homogeneity of the included studies, future meta‐analyses including a larger number of studies will be important for further assessing the robustness of our findings.^34^ Second, while the included studies used fMRI tasks designed to elicit inhibitory control, they may not fully capture the complexity of inhibitory processes engaged during therapy or in the real‐world environment. Developing tasks that more accurately reflect the cognitive demands of psychotherapy sessions would be an important next step. Furthermore, the majority of the studies included in this meta‐analysis (14 out of 15, that is 93.3%) are randomized controlled trials (RCTs). The remaining study demonstrated that the reported neural changes were significantly correlated with clinical symptom improvement. This statistical relationship suggests that the brain changes are closely linked to the therapeutic process (See details in Supplementary Table S1).

Moreover, the relationship between psychotherapy‐induced neural changes and clinical outcomes requires further exploration. Longitudinal studies are needed to determine whether observed changes in the PFC and ACC can predict long‐term symptom improvement. Finally, the interplay between neural changes and molecular targets, such as those influenced by psychotropic drugs, warrants investigation. Advanced approaches, including multimodal imaging, could provide insights into how psychotherapy interacts with neurochemical systems. Our findings represent an initial step toward understanding the shared mechanisms of action between psychotherapy and psychopharmacology, underscoring the potential of psychotherapy to induce measurable changes in brain activity, particularly within circuits crucial for inhibitory control. Moreover, elucidating the neural effects of psychotherapy can inform the development of complementary interventions, such as neuromodulation techniques or cognitive training protocols, aimed at further augmenting therapy‐induced changes.

In conclusion, this study contributes to the increasing evidence that psychotherapy engages and modifies the activation of key brain regions, particularly the PFC‐ACC network involved in inhibitory control. These findings mark a step forward in connecting psychological theory with neurobiological mechanisms, paving the way for a more integrative approach to mental health care.

## Author contributions

Conceptualization, G.G., F.G. and M.P.V.; methodology, G.G., O.O., V.B and FG.; formal analysis, G.G. and O.O., VB; data curation, O.O. V.B. writing—original draft preparation, G.G. and O.O.; writing—review and editing, M.P.V., G.G., V.R. All authors have read and agreed to the published version of the manuscript.

## Disclosure

The authors declare no conflict of interest.

## Supporting information


**Table S1.** Description of the included studies.

**Data S1.** PRISMA 2020 Checklist.

**Data S2.** PRISMA 2020 for Abstract Checklist.

## Data Availability

The data that support the findings of this study are available on request from the corresponding author. The data are not publicly available due to privacy or ethical restrictions.
