# Supplementary material for: Psychotherapy and inhibitory control: Insights from fMRI research
Source: Psychiatry Clin Neurosci. 2026 Mar 26;80(6):502–9. doi: 10.1111/pcn.70051 (PMC13244584; doi:10.1111/pcn.70051)
Supplement: Supplementary file 1 — Table S1. Description of the included studies. [file PCN-80-502-s002.pdf]

	DOI	Name of the Paper	Authors<br>Year	Task Description	Participant<br>Age<br>F/M	Condition<br>Treatment type and duration	Clinical Improvement<br>Inhibitory Control improvement	Analyzed Source*, Contrast	Study Design	fMRI Parameters	Stereotaxic Space<br>Data Processing Tool
**1**	10.1016/j.bpsc.2017.12.006	Cognitive Behavioral Therapy Is Associated With Enhanced Cognitive Control Network Activity in Major Depression and Posttraumatic Stress Disorder	Yang et al.<br>2018	Go/Nogo with Emotional Conflict Task<br>This task required participants to pay attention to either houses or fearful/neutral faces presented in a target axis (horizontal or vertical, randomized) while ignoring the stimuli presented in the other axis.	N=31<br>33,7<br>29/2	Major depression and post-traumatic stress disorder<br>CBT, 12 weeks	Improvement<br>Improvement	Table S1, (nogo vs go) x (pre-treatment vs post-treatment)	RCT	TR: 2200 ms<br>TE: 25 ms<br>Flip Angle: 90°<br>FOV: 205 mm<br>Number of Slices: 39<br>Voxel Size: 3.2 × 3.2 × 3.2 mm<br>Smoothing: 6 mm FWHM	MNI<br>FSL
**2**	10.1016/j.nicl.2020.102460	Stable inhibition-related inferior frontal hypoactivation and fronto-limbic hyperconnectivity in obsessive-compulsive disorder after concentrated exposure therapy	Lillevik Thorsen et al.<br>2020	Stop Signal Task<br>This task required responding to arrow directions (left/right) during go-trials as quickly and accurately as possible. Go-trials were pseudo-randomly intermixed with stop-trials, where a cross overlay signaled participants to withhold their response. The stop-signal delay was dynamically adjusted via a staircase tracking mechanism to maintain approximately 50% inhibition accuracy.	N=41<br>30,5<br>Not reported	Obsessive-compulsive disorder<br>CBT, 12 weeks	Improvement<br>Not significant improvement	Table S10, (nogo vs go) x (pre-treatment vs post-treatment)	RCT	TR: 2100 ms<br>TE: 30 ms<br>Flip Angle: 80°<br>FOV: 22 mm<br>Number of Slices: 34<br>Voxel Size: 3.44 × 3.44 × 3 mm<br>Smoothing: 8 mm FWHM	MNI<br>SPM12
**3**	10.1037/adb0000298	Functional neural changes following behavioral therapies and disulfiram for cocaine dependence	DeVito et al.<br>2017	Stroop Task Behavior<br>This task required participants to name the ink color of color-words presented in either congruent (e.g., “RED” in red ink) or incongruent (e.g., “RED” in blue ink) conditions. Participants were instructed to ignore the written word and respond only to the ink color as quickly and accurately as possible.	N=26<br>40.27<br>10/16	Cocaine dependence<br>CBT, 12 weeks	Improvement<br>Not Reported	Table 3, (incongruent vs congruent) x (pre-treatment vs post-treatment)	RCT	TR: 2280 ms<br>TE: 50 ms<br>Flip Angle: 90°<br>FOV: 192 mm x 192 mm<br>Number of Slices: 27<br>Voxel Size: 3 x 3 x 4 mm<br>Smoothing: Not reported	MNI<br>SPM 8
**4**	10.1176/appi.ajp.2013.12111484	Neural substrates of treatment response to cognitive-behavioral therapy in panic disorder with agoraphobia	Lueken et al.<br>2013	Inhibition Contrast extrapolated from the Fear-Conditioning Task<br>This task used a fear-conditioning method with three parts: familiarization, acquisition, and extinction. Participants saw colored shapes; one was a reinforced conditioned stimulus (CS+) paired with an unpleasant sound (US), and the other was a non-reinforced conditioned stimulus (CS–) that signaled safety. During the acquisition phase, the sound followed the CS+ 50% of the time. To measure learning accurately, the analysis only used trials where the CS+ appeared without the sound	N=42<br>34.83<br>29/13	Panic disorder<br>CBT, 12 weeks	Improvement<br>Not significant improvement	Table 2, (CS+ vs CS-) x (pre-treatment vs post-treatment)	RCT	TR: 2000 ms<br>TE: 30 ms<br>Flip Angle: 9°<br>FOV: 230 mm<br>Number of Slices: 30<br>Voxel Size: 3.6 × 3.6 × 3.8 mm<br>Smoothing: 12 mm FWHM	MNI<br>SPM5

**5**	10.1111/pcn.12357	Frontolimbic neural circuit changes in emotional processing and inhibitory control associated with clinical improvement following transference-focused psychotherapy in borderline personality disorder	Perez et al.<br>2016	Emotional Linguistic Go/No-go Task<br>This task required participants to perform a right-index-finger button-press immediately after (silently) reading a word appearing in normal font (go trial) and to inhibit this response after reading a word in italicized font (no-go trial). Button-press responses and reaction times were recorded.	N=10<br>27.8<br>10/0	Borderline personality disorder<br>TFP, (twice-weekly) approximately 9-10 months	Improvement<br>Not Reported	Table S3, (nogo vs go) x (pre-treatment vs post-treatment)	Longitudinal study, no control group	TR: 1200 ms<br>TE: 30 ms<br>Flip Angle: 70°<br>FOV: 240 mm<br>Number of Slices: 21<br>Voxel Size: 3.75 × 3.75 × 5 mm<br>Smoothing: 7.5 mm FWHM	MNI<br>SPM
**6**	10.1016/j.psychresns.2016.02.007	Brain effects of computer-assisted cognitive remediation therapy in anorexia nervosa: A pilot fMRI study	Brockmeyer et al.<br>2016	Go/No-go with Visual Target-Detection Task<br>This task used a vehicle classification system where participants saw different car images. They had to press one button for 'standard' cars and another button for 'target' vehicles (new cars). When they saw a 'non-target' vehicle (the target from the previous round), they had to avoid pressing any button. Each correct target was rewarded with 0.50 EUR, and participants received feedback on their earnings after each run.	N=12<br>22.82<br>Not reported	Anorexia nervosa<br>CRT, 3 weeks	Not Reported<br>Improvement	Table2, (nogo vs go) x (pre-treatment vs post-treatment)	RCT	TR: 2280 ms<br>TE: 50 ms<br>Flip Angle: 90°<br>FOV: 192 x 192 mm<br>Number of Slices: 27 slices<br>Voxel Size: 3 x 3 x 4 mm<br>Smoothing: 8 mm FWHM	MNI<br>SPM 8
**7**	10.1017/S0033291712000499	Treatment effects on insular and anterior cingulate cortex activation during classic and emotional Stroop interference in child abuse-related complex post-traumatic stress disorder	Thomaes et al.<br>2012	Stroop task<br>This task consisted of randomized blocks featuring six word categories: congruent words (e.g., 'red' in red), incongruent words (e.g., 'red' in blue), trauma words (e.g., 'rape' in red), general negative words (e.g., 'illness' in red), and two series of neutral words (e.g., 'table' in red). Participants were asked to respond as fast as possible to the ink color. Before the scanning session, patients practiced the task using only congruent and neutral words both outside and inside the scanner.	N=9<br>35.2<br>9/0	Post-traumatic stress disorder<br>Psycho-Educational and Cognitive Behavioural Stabilizing Group Treatment, 20 weeks	Improvement<br>Improvement	Table 5, (incongruent vs congruent) x (pre-treatment vs post-treatment)	RCT	TR: 2920 ms<br>TE: 45 ms<br>Flip Angle: 90°<br>FOV: 192 x 192 mm<br>Number of Slices: 35<br>Voxel Size: 3 x 3 x 3 mm<br>Smoothing: 6 mm FWHM	MNI<br>SPM5
**8**	10.1016/j.psychresns.2010.06.005	Changes of brain activation pre- post short-term psychodynamic inpatient psychotherapy: an fMRI study of panic disorder patients	Beutel et al.<br>2010	Go/No go task<br>This task required participants to perform a right-index-finger button press immediately after reading a word in normal font (Go trial) and to inhibit this response when the word appeared in italics (No-Go trial). Response accuracy and reaction times were recorded to measure inhibitory control.	N=27<br>30<br>15/12	Panic disorder<br>Short-Term Psychodynamic inpatient treatment, 4 weeks	Improvement<br>Not significant improvement	Table 5, (nogo vs go) x (pre-treatment vs post-treatment)	RCT	TR: 3000 ms<br>TE: 60 ms<br>Flip Angle: 90°<br>FOV: 190 mm<br>Number of Slices: 30<br>Voxel Size: 3 x 3 x 5 mm<br>Smoothing: 9 mm FWHM	MNI<br>SPM
**9**	10.1017/s0007125000161872	Effects on the brain of a psychological treatment: cognitive remediation therapy: functional magnetic resonance imaging in schizophrenia	Wykes et al.<br>2002	Stroop Neuropsychological Screening Test	N=12<br>35,5<br>0/12	Schizophrenia<br>CRT, 12 weeks	Not significant improvement<br>Not significant improvement	Table 2, (incongruent vs congruent) x (pre-treatment vs post-treatment)	RCT	TR: 3000 ms<br>TE: 40 ms<br>Flip Angle: Not reported<br>FOV: Not reported<br>Number of Slices: 10<br>Voxel Size: Not reported<br>Smoothing: Not reported	Talairach<br>ANMR

**10**	10.1016/j.addbeh.2018.09.005	fMRI Stroop and behavioral treatment for cocaine-dependence: Preliminary findings in methadone-maintained individuals	DeVito et al. / 2019	Stroop Task<br>This task required participants to name the ink color of color-words presented in congruent (e.g., 'RED' in red) or incongruent (e.g., 'RED' in blue) conditions. Participants performed six task runs, each including seven pseudo-randomized incongruent trials. Verbal response times were recorded via microphone, while researchers manually tracked errors. Practice runs were completed prior to scanning, and performance was recorded during subsequent runs to assess interference effects.	N=10 / 39,6 / 7/3	Cocaine-dependence / CBT, 8 weeks	Improvement / Not significant improvement	Table 1, (incongruent vs congruent) x (pre-treatment vs post-treatment)	RCT	TR: 1500 ms<br>TE: 27 ms<br>Flip Angle: 60°<br>FOV: 220 x 220 mm<br>Number of Slices: 25<br>Voxel Size: 3 x 3 x 3 mm<br>Smoothing: 6 mm FWHM	MNI / SPM5
**11**	10.1016/j.drugalcdep.2011.10.002	A preliminary study of the neural effects of behavioral therapy for substance use disorders	DeVito et al. / 2012	Stroop Task<br>This task required participants to perform six runs of an event-related fMRI Stroop paradigm by silently naming the ink color of congruent (e.g., 'red' in red) or incongruent (e.g., 'red' in blue) color-word pairs. Each run included seven incongruent events, which were presented pseudo-randomly every 13–16 congruent stimuli. This design was used to measure the brain's response to color-word interference during functional imaging.	N=12 / 37.2 / 5/7	Substance use disorders / CBT and TAU, 8 weeks	Improvement / Improvement	Table 5, (incongruent vs congruent) x (pre-treatment vs post-treatment)	RCT	TR: 1500 ms<br>TE: 27 ms<br>Flip Angle: 60°<br>FOV: 22 x 22 cm<br>Number of Slices: 25<br>Voxel Size: 3.4 x 3.4 x 5 mm<br>Smoothing: 9 mm FWHM	MNI / SPM2
**12**	10.1016/j.psychres.2013.07.007	Modulation of fronto-limbic activity by the psychoeducation in euthymic bipolar patients. A functional MRI study	Favre et al. / 2013	Word-Face Emotional Stroop Task<br>This task used grayscale faces of various ethnic origins (African, Asian, Caucasian, and Hispanic) expressing happy, fearful, or neutral emotions. Emotional words ("JOIE" or "PEUR") were superimposed on these faces to create congruent or incongruent stimuli, while neutral faces were paired with the word "RIEN" as controls. Participants were required to identify the facial expression as quickly and accurately as possible while ignoring the written word. Responses were made using three buttons with the dominant hand to distinguish between happy, fearful, and neutral expressions.	N=26 / Not reported / Not reported	Bipolar patients / The Psycho-education Program, 12 weeks	Improvement / Improvement	Table 3, (incongruent vs congruent) x (pre-treatment vs post-treatment)	RCT	TR: 3000 ms<br>TE: 30 ms<br>Flip Angle: 77°<br>FOV: 216 x 216 mm<br>Number of Slices: 47 axial<br>Voxel Size: 3 x 3 x 3 mm<br>Smoothing: 8 mm FWHM	MNI / SPM8
**13**	10.1155/2024/6687657	Pharmacological and Psychological Treatment Have Common and Specific Effects on Brain Activity in Obsessive-Compulsive Disorder	Van der Straten et al. / 2024	Stop Signal Task<br>This task consisted of 180 go trials and 40 stop trials. The time delay between the go cue and the stop signal (Stop Signal Delay) was dynamically adjusted using a staircase procedure to maintain an average inhibition success rate of approximately 50%. This allowed for the calculation of the stop signal reaction time as a measure of inhibitory control.	N=34 / 30,62 / 17/17	Obsessive-compulsive disorder / CBT, 16 weeks	Improvement / Not Reported	Table 2, (nogo vs go) x (pre-treatment vs post-treatment)	RCT	TR: 2375 ms<br>TE: 9, 26, and 44 ms (3 echos)<br>Flip Angle: 76°<br>FOV: 224 x 122 x 224 mm<br>Number of Slices: 37<br>Voxel Size: 2 mm<br>Smoothing: 8 mm FWHM	MNI / SPM12

**14**	10.1016/j.biopsych.2004.12.039.	Brain activation of patients with obsessive-compulsive disorder during neuropsychological and symptom provocation tasks before and after symptom improvement: a functional magnetic resonance imaging study.	Nakao et al.<br>2005	Stroop Task<br>This task required participants to name the ink color of Chinese characters while ignoring their semantic meaning (e.g., the character for 'red' printed in blue ink). Under this condition, subjects must attend to the color value while suppressing the conflicting semantic value, creating a mismatch that tests cognitive control. During fMRI scanning, the naming task was performed silently to minimize head movement and ensure data quality.	N=10<br>32.4<br>6/4	Obsessive-compulsive disorder<br>CBT, 12 weeks	Improvement<br>Not significant improvement	Table 8&9, (incongruent vs congruent) x (pre-treatment vs post-treatment)	RCT	TR: 4000 ms<br>TE: 50 ms<br>Flip Angle: 90°<br>FOV: 230 mm<br>Number of Slices: 32<br>Voxel Size: 3.6 x 3.6 x 3 mm<br>Smoothing: 12 mm FWHM	Talairach<br>SPM99
**15**	10.1016/j.psychresns.2007.11.001.	Functional MRI study of brain activation alterations in patients with obsessive-compulsive disorder after symptom improvement.	Nabeyama et al.<br>2008	Stroop Task<br>This task used a Chinese character version of the Stroop paradigm, featuring both control and interference conditions. In the control condition, participants silently named the ink color when it matched the character's meaning. In the interference condition, they named the ink color of characters printed in a conflicting color. To ensure consistent performance during fMRI scanning, participants completed thorough training before the session.	N=11<br>32.4<br>7/4	Obsessive-compulsive disorder<br>CBT, 12 weeks	Improvement<br>Improvement	Table 6, (incongruent vs congruent) x (pre-treatment vs post-treatment)	RCT	TR: 4000 ms<br>TE: 50 ms<br>Flip Angle: 90°<br>FOV: 230 mm<br>Number of Slices: 32 axial<br>Voxel Size: 3.6 x 3.6 x 3 mm<br>Smoothing: 12 mm FWHM	Talairach<br>SPM99

N=Number of participants, **F**=Female, **M**=Male, **CBT**=Cognitive Behavioral Therapy, **TFP=** Transference-Focused Psychotherapy, **CRT=** Cognitive Remediation Therapy, **TAU=** Treatment As Usual, **CS+**=conditioned stimulus associated with the unconditioned stimulus, **CS-** =conditioned stimulus associated with the unconditioned stimulus, **RCT**=Randomized Controlled Trial, **MNI=**Montreal Neurological Institute, **FOV=** Field of View, **TR=** Repetition Time, **TE=** Echo Time, **FSL**=FMRIB's Software Library, **FMRIB=** Functional Magnetic Resonance Imaging of the Brain, **FWHM=**Full Width at Half Maximum, **SPM=** Statistical Parametric Mapping, **ANMR=** Advanced Nuclear Magnetic Resonance

*Analyzed sources were filtered by subheading
